# The mechanics and interactions of electrically sensitive mechanoreceptive hair arrays of arthropods

**DOI:** 10.1098/rsif.2022.0053

**Published:** 2022-03-23

**Authors:** Ryan A. Palmer, Isaac V. Chenchiah, Daniel Robert

**Affiliations:** ^1^ School of Biological Sciences, University of Bristol, Life Sciences Building, 24 Tyndall Avenue, Bristol BS8 1TQ, UK; ^2^ School of Mathematics, University of Bristol, Fry Building, Woodland Road, Bristol BS8 1UG, UK

**Keywords:** mechanoreceptor, mechanosensory, electroreception, electrostatic interaction, sensory hairs, arthropods

## Abstract

Recent investigations highlight the possibility of electroreception within arthropods through charged mechanosensory hairs. This discovery raises questions about the influence of electrostatic interaction between hairs and surrounding electrical fields within this sensory modality. Here, we investigate these questions by studying electrostatic coupling in arrays of hairs. We establish the notion of sensitivity contours that indicate regions within which point charges deflect hairs beyond a given threshold. We then examine how the contour’s shape and size and the overall hair behaviour change in response to variations in the coupling between hairs. This investigation unveils synergistic behaviours whereby the sensitivity of hairs is enhanced or inhibited by neighbouring hairs. The hair spacing and ratio of a system’s electrical parameters to its mechanical parameters influence this behaviour. Our results indicate that electrostatic interaction between hairs leads to emergent sensory properties for biologically relevant parameter values. The analysis raises new questions around the impact of electrostatic interaction on the current understanding of sensory hair processes, such as acoustic sensing, unveiling new sensory capabilities within electroreception such as amplification of hair sensitivity and location detection of charges in the environment.

## 1. Introduction

Mechanoreception using deflectable hairs is a fundamental sensory process across Arthropoda, most notably among insects, arachnids and crustaceans. Mechanosensory hairs display remarkable morphological diversity, even within a single specimen, and have a wide range of functional properties. Thus, these hairs enable an animal to carry out a variety of sensory tasks (e.g. hearing, fluid flow sensing and proprioception; see [1] for a comprehensive review) and non-sensory tasks (e.g. thermal insulation [2,3] and pollen grain capture [4,5]).

The adaptation of mechanosensory filiform hairs to respond to acoustic cues and fluid flows has been extensively studied and described for insects and spiders through mathematical modelling and empirical studies [1,6–16]. However, only recently filiform hairs have been shown to respond to ecologically relevant weak electric fields, with evidence found in honeybees [17], bumblebees [18,19] and spiders [20]. Particular attention has been paid to the interaction of bees with floral electric fields and global atmospheric potential gradients [5,13,18]. The capacity to detect electrical fields is deemed possible owing to insects and spiders becoming electrically charged as they interact with their environment, resulting in the filiform hairs becoming sensitive to electrical stimuli. For a broad survey of the literature on the ecology of electricity and electroreception, we refer readers to [21].

Mechanosensory hairs are often organized in arrays, forming either a sparse cover or dense fur. When electrically charged, a mutual influence on a hair’s sensory response is possible through Coulomb interaction with neighbouring hairs. This leads to questions about electromechanical sensing through arrays of hairs, and over a whole body. Thus far, studies on electrical sensing have solely focused on the mechanisms of individual hairs [13,22]. By contrast, there are several studies of mechanosensory hair responses to aerodynamic and acoustic stimuli as singlets, arrays and whole systems (as singlets: spider trichobothria [23–26], cricket cercal hairs [27,28]; as arrays: spider trichobothria [10], cricket cercal hairs [12,29]; as whole systems: cricket cercal sensors [30,31]).

Aerodynamic studies of hair arrays have considered viscous-mediated hair–hair coupling. First suggested in [23] for spider trichobothria and examined further in [10], it has been mathematically and experimentally shown that the mechanical hair response to aerodynamic stimuli can be influenced by neighbouring hairs through viscous effects and each hair’s disruptive effect on the flow. Similar results have been reported for cricket cercal hairs; see, for example, [12,31–33]. Hence, the response of a hair in an array is influenced by neighbouring hairs through viscous-mediated coupling (to varying degrees). Such coupling may enhance or reduce the response in another hair [10].

An electrical equivalent, namely electrostatic coupling, can have similar effects. Each charged hair within an array generates an electrical field that interacts with the wider electrical field of the surrounding environment (i.e. electrical fields external to the array and the electrical fields from the other hairs). Therefore, the response of a hair is influenced by neighbouring hairs through Coulomb interactions (to varying degrees).

We are further motivated by the morphological observation that arrays of filiform hairs exhibit significant variation in their proximity, spatial organization and morphological distribution from species to species. Arrays can range from a few hairs of varying length arranged in rows, such as the trichobothria on the legs of the spider *Cupiennius salei* [25], to dense mats, such as the cercus of the cricket *Gryllus bimaculatus* [27,34], or to regular arrangements, such as those found on the cercus of the cockroach *Periplaneta americana* [35]. The density of hairs covering different pollinating insect species has been carefully classified for 109 species and showed great diversity even among closely related species [36]. Indeed, hair density and organization can vary greatly over the cuticular surface area of a single insect, depending on the body part considered [36]. In considering information acquisition, questions arise about why hair coverage (of mechanosensory hairs in particular) varies greatly and what the adaptive value is of varying hair density and distribution [37]. Here, we begin to explore the functional implications of the density of hair coverage using electroreception as a sensory modality.

In this paper, we consider the mechanical influence of electrostatic coupling within hair arrays and the properties that determine its strength. In §2, we summarize the basic theory used to model electrical sensing in arrays of *N* charged hairs. The primary computational examples focus on systems of two hairs. In §3, we discuss and calculate several metrics of electrostatic coupling in multi-hair systems to assess when the coupling is enhancing or inhibiting. Finally, in §4, we discuss the wider mechanical and biological implications of our results. (For reference, a table of the parameters and their units is provided in electronic supplementary material, A. Furthermore, an introduction to the basic concepts is provided through an analysis of a single-hair system in electronic supplementary material, B)

## 2. Electrostatic sensing with *N* hairs

### 2.1. Equations of motion

#### 2.1.1. Mechanosensory hairs as harmonic oscillators

A mechanosensory hair is typically modelled as an inverted linear pendulum, with the hair acting as a rigid rod [6,9,11,22]. Hair motion is influenced by several of its features, such as length, shape and the properties of the viscoelastic socket membrane [38,39], which lead to the oscillator parameters *S* (kg m^2^ s^−2^) (the restoring/spring constant), *R* (kg m^2^ s^−1^) (the torsional resistance/damping constant) and *I* (kg m^2^) (the moment of inertia) [11].

When an external force **F** (kg m s^−2^) acts at a position **r** (m) along the hair a resultant torque ***τ*** (kg m^2^ s^−2^) occurs and is related to the hair motion through

2.1 $$-\tau =I\ddot{\theta }(t)+R\dot{\theta }(t)+S\theta (t),$$

where *τ* = |***τ***|, *θ* (rad) is the angular deflection of the hair (i.e. the angle between the hair’s perturbed position and its resting position). The dots indicate time derivatives.

**Figure 1.**
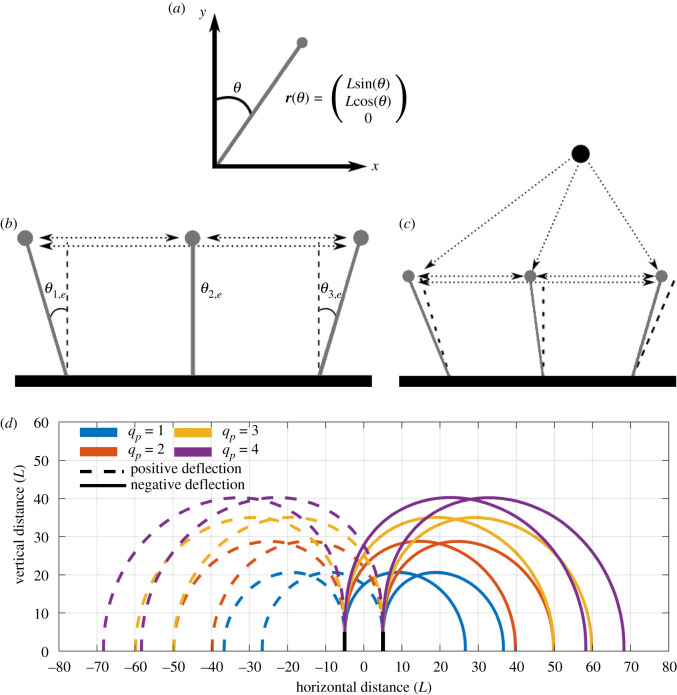
Diagrams of multiple hair mechanics. In (*b*,*c*), dashed lines indicate the equilibrium positions of the hairs. (*a*) Hair coordinate system (note that the *z*-axis points out of the page). (*b*) Force diagram of electrostatic interaction between hairs. (*c*) Force diagram of electrostatic interaction between hairs and an external point charge. (*d*) Solution to (2.9) for *q*_*p*_ = 1, 2, 3, 4, *K* = 1 and *δ* = 10. Contours show locations of the external point charge that deflect a hair by $\theta_{s}=0.001\;\mathrm{rad}$ from its equilibrium position.

The resultant torque is given by ***τ*** = **r** × **F**, where **r** is a vector for the hair position (as shown in figure 1*a*) and **F** is a force acting on the hair. We consider the motion of the hair within a plane (as is common within the aerodynamic literature, e.g. [11,12]). We therefore constrain hair motion to a plane with normal in the negative *z*-direction such that **F** = (*F*_*x*_, *F*_*y*_, 0).

In this work, we consider the quasi-static situation so that the terms $I\ddot{\theta }(t)$ and $R\dot{\theta }(t)$ are negligible. This approximation holds well for fixed-point charges and for slowly evolving systems. From this quasi-static approximation, a foundational understanding can be gained for the sensory mechanics and Coulomb interaction of charged hairs. This forms the basis for studying time-varying systems (with the inclusion of the above dynamic terms). This quasi-static analysis enables significant progress in understanding this complex and multi-faceted sensory phenomenon.

#### 2.1.2. Coulomb interactions between hairs

Consider *N* hairs distributed along an arbitrary curve. For simplicity, we assume the total charge hair *q*_*h*_ (*C*) is located at the hair tip, with *h* = 1, 2, …, *N*. The resting position of the hair tip is defined by the location of its base, **x**_*h*,0_ = (*x*_*h*,0_, *y*_*h*,0_, *z*_*h*,0_) (m), its resting angle, *θ*_*h*,0_ (rad) (measured clockwise about the *z*-axis) and its length, *L*_*h*_ (m). We consider *z*_*h*,0_ = 0 so that the hairs and their motion are co-planar. The location of the *h*th hair tip at an angle of deflection *θ*_*h*_ (rad) is given by

2.2 $$x_{h}=(x_{h},y_{h},z_{h})=(x_{h,0}+L_{h}\sin\theta_{h},\;y_{h,0}+L_{h}\cos\theta_{h},\;0).$$

Defining **r**_*h*_ = (*L*_*h*_ sin*θ*_*h*_, *L*_*h*_ cos*θ*_*h*_, 0), the torque is given by

2.3 $$\tau_{h}=|r_{h}\times F_{h}|=-L_{h}\cos(\theta_{h})F_{h,x}+L_{h}\sin(\theta_{h})F_{h,y}.$$

Each hair deflects from its resting position to an equilibrium position owing to Coulomb interaction between all the hairs, presented by a force diagram in figure 1*b*. This equilibrium position is calculated first and described by *θ*_*h*,*e*_, **x**_*h*,*e*_ = (*x*_*h*,*e*_, *y*_*h*,*e*_, *z*_*h*,*e*_) for each hair tip (noting that *z*_*h*,*e*_ = 0).

The Coulomb force on the *h*th hair due to the *N* − 1 other hairs is given by

2.4 $$F_{h,e}=\frac{q_{h}}{k_{e}}\sum_{i\neq h}^{N}q_{i}\frac{x_{h,e}-x_{i,e}}{{\left|x_{h,e}-x_{i,e}\right|}^{3}},$$

where *k*_*e*_ ≈ 8.988 × 10^9^ (Nm^2^ C^−2^) is the Coulomb constant. From (2.1) and (2.3), we have

2.5 $$S_{h}\theta_{h,e}=L_{h}\cos(\theta_{h})F_{h,x}-L_{h}\sin(\theta_{h})F_{h,y}.$$

Thus, the equilibrium position of the *h*th hair is given by

2.6 $$\theta_{h,e}=\frac{q_{h}}{k_{e}}\frac{L_{h}}{S_{h}}\sum_{i\neq h}q_{j}\frac{\left(x_{h,e}-x_{i,e})\cos(\theta_{h,e})-(y_{h,e}-y_{i,e})\sin(\theta_{h,e}\right)}{{\left({\left(x_{h,e}-x_{i,e}\right)}^{2}+{\left(y_{h,e}-y_{i,e}\right)}^{2}\right)}^{3/2}},$$

producing *N* simultaneous equations.

#### 2.1.3. External point charge

A point charge is described by (*x*_*p*_, *y*_*p*_, *z*_*p*_) (m), *q*_*p*_ (C) and is fixed, i.e. the Coulomb force generated by the hair does not move the point charge. We consider *z*_*p*_ = 0 so that the point charge and hairs are co-planar. A force diagram for the hair deflections due to the point charge is displayed in figure 1*c*. Following from (2.1) and (2.3), the angular deflection of the *h*th hair due to the presence of *N* − 1 other hairs and an external point charge is given by

2.7 $$\begin{array}{rl} \theta_{h} & =\frac{q_{h}}{k_{e}}\frac{L_{h}}{S_{h}}(q_{p}\frac{\left(x_{h}-x_{p})\cos(\theta_{h})-(y_{h}-y_{p})\sin(\theta_{h}\right)}{{\left({\left(x_{h}-x_{p}\right)}^{2}+{\left(y_{h}-y_{p}\right)}^{2}\right)}^{3/2}}\\ & \;+\sum_{i\neq h}q_{i}\frac{\left(x_{h}-x_{i})\cos(\theta_{h})-(y_{h}-y_{i})\sin(\theta_{h}\right)}{{\left({\left(x_{h}-x_{i}\right)}^{2}+{\left(y_{h}-y_{i}\right)}^{2}\right)}^{3/2}}). \end{array}$$

To aid the analysis of (2.7), we non-dimensionalize the system to reduce the number of parameters. Consider the scaling

2.8 $$(L_{h},S_{h},x_{h},y_{h},q_{h},x_{p},y_{p},\;q_{p})=(L{\tilde{L}}_{h},S{\tilde{S}}_{h},L{\tilde{x}}_{h},L{\tilde{y}}_{h},q{\tilde{q}}_{h},L{\tilde{x}}_{p},L{\tilde{y}}_{p},q{\tilde{q}}_{p}),$$

where the tilde notation denotes the non-dimensional values. Here, *L* is the typical hair length, *S* is the typical spring constant and *q* is the typical hair charge. It is expected that ${\tilde{L}}_{i},{\tilde{S}}_{i},{\tilde{q}}_{i}$ remain of order unity for *i* = 1, 2, …, *N*, *j*, while ${\tilde{q}}_{p}$, ${\tilde{x}}_{p}$ and ${\tilde{y}}_{p}$ may vary by several orders of magnitude. Thus, (2.7) becomes

2.9 $$\begin{array}{rl} \theta_{h} & =K\frac{{\tilde{q}}_{h}{\tilde{L}}_{h}}{{\tilde{S}}_{h}}({\tilde{q}}_{p}\frac{\left({\tilde{x}}_{h}-{\tilde{x}}_{p})\cos(\theta_{h})-({\tilde{y}}_{h}-{\tilde{y}}_{p})\sin(\theta_{h}\right)}{{\left({\left({\tilde{x}}_{h}-{\tilde{x}}_{p}\right)}^{2}+{\left({\tilde{y}}_{h}-{\tilde{y}}_{p}\right)}^{2}\right)}^{3/2}}\\ & \;+\sum_{i\neq h}{\tilde{q}}_{i}\frac{\left({\tilde{x}}_{h}-{\tilde{x}}_{i})\cos(\theta_{h})-({\tilde{y}}_{h}-{\tilde{y}}_{i})\sin(\theta_{h}\right)}{{\left({\left({\tilde{x}}_{h}-{\tilde{x}}_{i}\right)}^{2}+{\left({\tilde{y}}_{h}-{\tilde{y}}_{i}\right)}^{2}\right)}^{3/2}}), \end{array}$$

where

$$K=\frac{q^{2}}{k_{e}}\frac{1}{LS},$$

which is the ratio of the electrical parameters of the hair system to its mechanical parameters. We denote *K* as the parameter of electromechanical sensitivity.

Overall, the motion of the hairs is governed by three parameters: *K*, ${\tilde{q}}_{p}$ and the distance between the hair bases, denoted *δ*.

### 2.2. Biological parameter values

Biologically relevant values of *K* depend on the typical: hair length, *L*; spring constant, *S*; hair charge, *q*_*h*_; and point charge, *q*_*p*_. Conservative assumptions for these parameters are outlined below; nonetheless, through the non-dimensional analysis the exact parameter values are not required since we are concerned with characterizing the behaviour of the system for different ratios of them.

#### 2.2.1. Hair length

The hair length is expected to be of the order of millimetres, so *L* = *O*(10^−3^), which is typical for a wide range of arthropods and pollinators [36].

#### 2.2.2. Spring constant

Generally, the spring constant of mechanoreceptor hairs is unknown for different arthropods. However, within the literature allometric equations for *S* (as a function of *L*) have been devised for specific animals. Two examples are spider MeD1 trichobothria (1.272 × 10^−5^ *L*^2.030^) [11] and cricket cercal hairs (1.944 × 10^−6^ *L*^1.67^) [9], which both give *S* = *O*(10^−11^) for *L* = *O*(10^−3^). Therefore, conservatively, we consider *S* between 10^−12^ and 10^−10^, which is reasonable for a range of hairs [40].

#### 2.2.3. Hair charge

There are still unanswered questions about the magnitude and distribution of charge on a single hair. However, we can calculate illustrative bounds as follows.

For an upper bound, consider a cylindrical hair and suppose that the total charge is a function of the total surface charge of the hair (irrespective of charge distribution) such that *q*_*h*_ = *πdLσ*, where *d* (m) is the hair diameter and *σ* (Cm^−2^) is the surface charge density. From [13], *σ* may be calculated at the limit of dielectric breakdown to give *σ* = 10^−4^ (Cm^−2^). For the diameter of a hair, we may again use allometric equations for spider MeD1 trichobothria (6.343 × 10^−5^ *L*^0.3063^) [11] and cricket cercal hairs (8.34 × 10^−4^ *L*^0.67^) [9], both giving a diameter of the order of 10^−6^. Altogether, an upper bound for the hair charge is *q*_*h*_ = *O*(10^−11^).

For a lower bound, in [5,18], the net charge of a bee was measured to be of the order of *pC* = 10^−12^ (C). Thus, considering the hair charge to be an order of magnitude smaller, we gain a lower bound of *q*_*h*_ = 10^−13^ (C).

Regarding the point charge, we assume that *q*_*p*_ is within an order of magnitude of the hair’s charge. In general, the choice of *q*_*p*_ mainly determines the range of the contours and therefore acts as a scaling parameter in this system.

#### 2.2.4. Electromechanical constant *K*

**Figure 2.**
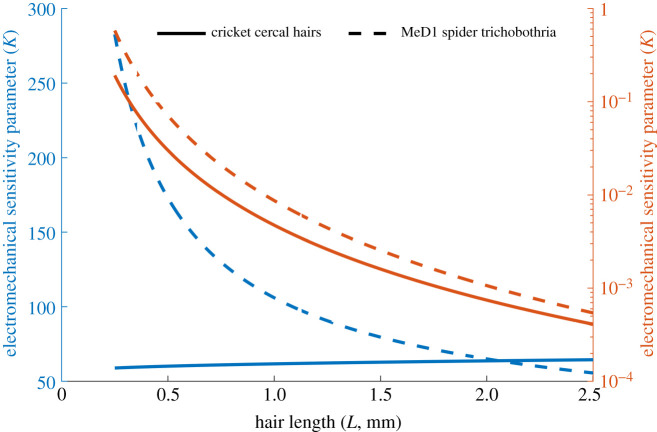
The electromechanical sensitivity parameter *K* for cricket cercal hairs (solid) and spider MeD1 trichobothria (dashed). Blue represents *q*_*h*_ ∼ 10^−11^ and red represents *q*_*h*_ ∼ 10^−13^.

Considering spider MeD1 trichobothria and cricket cercal hairs, we can calculate biologically relevant and physically feasible values of *K* using our estimated upper and lower bounds of *q*_*h*_, presented in figure 2. These results suggest the possibility for *K* to vary between 0.001 and 100, which covers a range of feasible scenarios for general biological systems. Thus, in the analysis presented we characterize the dynamics of mechanoreceptor hair systems for values of *K* in this range.

#### 2.2.5. Hair spacing

In [36], hair coverage for several pollinator species is found to be 100–1000 (hairs/mm^2^), giving 10–30 (hairs/mm) in one dimension. For *L* = *O*(10^−3^), *δ* is between 0.1 and 0.03 hair lengths. We therefore examine values of *δ* between 0.01 and 10 to cover the range of biologically relevant spacings and to identify trends in dynamics for this broad range. (Hair spacing is typically scaled by hair diameter; however, the hairs considered here are diameterless since this parameter is not required in the present model. When temporal effects are considered, hair diameter will no longer be negligible and may be used to scale the spacing.)

#### 2.2.6. Deflection sensitivity threshold

The sensitivity threshold is chosen to be $\theta_{s}=0.001$ $\mathrm{rad}∼0.057^{\circ }$ following discussions within the literature for bees, spiders and crickets [5,11,28]. This threshold is within the order of magnitude of deflections that produce neural responses in cricket systems ($0.0032\;\mathrm{rad}∼0.02^{\circ }$) [28] and in spiders (for the trichobothria of *Cupiennius salei*, 0.0016-0.016$\;\mathrm{rad}∼0.01-0.1^{\circ }$) [14]. Indeed, this threshold is conservative when compared with the neural sensitivity of *Gryllus bimaculatus* cercal hairs (0.00032−0.00016 rad∼0.002− $0.001^{\circ }$) [41]. Mathematically, the choice of *θ*_*s*_ is arbitrary, if it remains small, owing to the non-dimensionality and linearity of the oscillator system. Thus, the results presented throughout the paper hold for different values of *θ*_*s*_ and cover the typical sensory mechanics for many biologically relevant deflections.

### 2.3. Sensitivity contours for two hairs

A central question we explore in this paper is: at what locations around a hair array does an external point charge deflect at least one of the hairs within the range of sensory sensitivity? The sensitivity contour for the *h*th hair tip is dependent on the Coulomb interaction with the external point charge and the other *N* − 1 hairs. Figure 1*d* illustrates the contours for two hairs spaced 10 hair lengths apart. Here and subsequently, the hair parameters for the two hairs are assumed to be the same and of order unity unless stated otherwise, with dashed curves indicating the point charge locations that induce positive deflections, and solid curves indicating locations that produce negative deflections.

The contours show the point charge locations that lead to deflections of $|\theta_{h}|\geq |\theta_{s}|=0.001\;\mathrm{rad}$ in either hair for four different values of ${\tilde{q}}_{p}$. If a smaller *θ*_*s*_ is considered, the range of the contours will increase, while larger values will decrease the contour’s range. For points on the contours *θ*_*h*_ = *θ*_*s*_. Points inside these curves lead to greater deflections and points outside lead to below-threshold deflections.

This figure highlights the presence of a sensory ‘blind spot’—the region above a hair, outside of the contours, where the distance at which a point charge can be detected is significantly reduced. Here, point charges must be much closer for the hair to deflect to the sensory minimum owing to the small perpendicular component of the Coulomb force. Another feature of interest is the larger horizontal range compared with the vertical range.

In this example, the two hairs act independently because of the large spacing and we see that the contours are isotropically doubled for *q*_*p*_ = 4 compared with *q*_*p*_ = 1, showing the square law present in Coulomb interactions. However, this relationship may breakdown as the hair–hair interaction increases. Therefore, the pertinent questions are: can the contours change as a result of the presence of neighbouring hairs? If yes, what parameters govern the strength of interaction between them? This is examined in the next section.

### 2.4. Numerical results for two identical hairs

**Figure 3.**
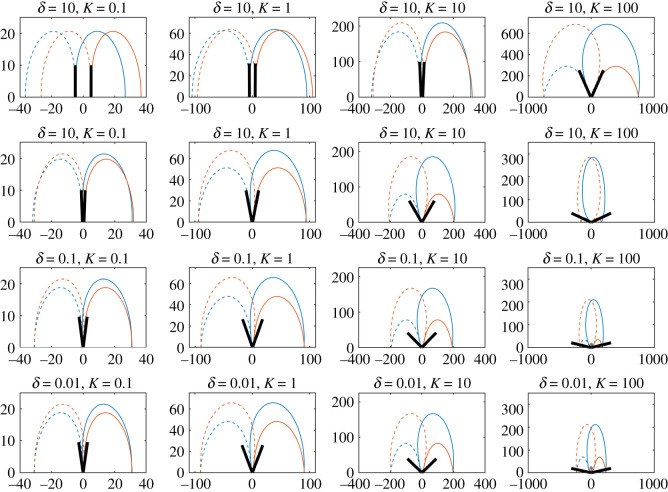
Sensitivity contours for two hairs from (2.9). Contours show locations of an external point charge *q*_*p*_ = 10 that deflect hairs by at least 0.001 rad from their equilibrium positions for *δ* = 10, 1, 0.1, 0.01 (from top to bottom) and *K* = 0.1, 1, 10, 100 (from left to right). The horizontal unit is *x*_*p*_/*L* and the vertical unit is *y*_*p*_/*L,* with both measured in hair lengths. Blue curves relate to the left hair and red curves relate to the right hair. The black lines show the equilibrium position of the hairs (not to scale).

The sensory contours of two hairs (same length and charge) and an external point charge *q*_*p*_ = 10 are displayed in figure 3, indicating the locations of an external point charge that induce positive or negative deflections of the hair from its equilibrium position by 0.001 rad. The resting angle is *θ*_*h*,0_ = 0, for *h* = 1, 2.

Starting with the top left plot where *δ* = 10 and *K* = 0.1, the two hairs act as two independent sensors owing to the large spacing between them and low electromechanical sensitivity (as governed by *K*). When *K* increases (moving from left to right) hair–hair interaction becomes more significant, even for the large spacing, as shown by the change in the contour’s shape and the larger equilibrium angles (as shown by the black lines). Similarly, stronger hair–hair interaction occurs for fixed (and small) values of *K* when the hair spacing diminishes (moving from top to bottom). For larger hair–hair interaction, the equilibrium positions of the hairs no longer bisect the contours for positive and negative deflections. In these regions, the hair–hair interaction is more influential than the presence of the point charge. When *K* = 100 and *δ* = 0.1, 0.01, numerical artefacts appear between the hairs and cannot be considered as valid physical behaviour.

Overall, variation in either *δ* or *K* readily affects the strength of hair–hair interaction with *δ* less influential on the hair coupling than *K*. In varying *δ* for a fixed *K*, the contours remain of a similar order of magnitude, while fixing *δ* and increasing *K* leads to larger contours up to a point.

For large *δ*, changing the magnitude of *K* varies the magnitude of the contours proportionally by $\sqrt{K}$. In some cases, when *δ* is small and the hair–hair interaction becomes significant (third row), the geometry and magnitude of the contours change such that this property is lost. This highlights that the isotropic property of the contours can break down as the hair–hair interaction increases.

**Figure 4.**
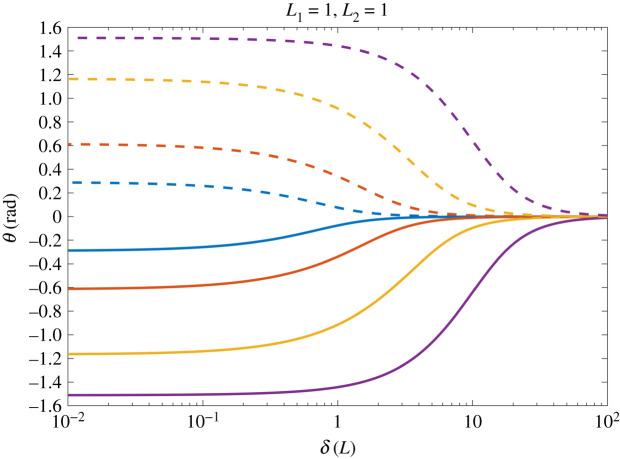
The effect of hair spacing *δ* on the equilibrium position of two hairs for *K* = 0.1, 1, 10, 100 (blue, red, yellow, purple, respectively).

Furthermore, there is indication of a limit as *δ* diminishes, for each given *K* (compare the plot for *δ* = 0.1, *K* = 10 with *δ* = 0.01, *K* = 10, and the plot for *δ* = 0.1, *K* = 100 with *δ* = 0.01, *K* = 100). This is verified by figure 4, where the equilibrium angles of the two hairs are presented for values of *δ* ∈ [10^−2^, 10^2^] and *K* = 0.1, 1, 10, 100. When the initial hair spacing *δ* diminishes the equilibrium angle between the two hairs tends to a limit dependent on *K* and is overall limited to a maximum of *π*/2. Thus, there is a point at which reducing the spacing has no further influence on the hair dynamics.

Overall, *K* determines the electromechanical sensitivity of the hairs (e.g. the distance over which significant deflections may occur) and *δ* modulates the level of interaction between the hairs within the band of sensitivity set by *K*. With the variation of these parameters, qualitatively different behaviours are seen in the hair–hair and hair–point charge interactions. Furthermore, the equilibrium angle of the hairs is a helpful metric for understanding the magnitude of hair–hair coupling. This will be discussed further in §4.

### 2.5. Analysis of *N* hair system

#### 2.5.1. Small equilibrium angle

In figure 4, the equilibrium positions remain close to the resting positions when *K* is small or *δ* is large. To understand this more generally, consider the scenario for *N* hairs where the equilibrium angles of the hairs are small, i.e. *θ*_*h*,*e*_ ≈ 0, sin*θ*_*h*,*e*_ ≈ 0 and cos*θ*_*h*,*e*_ ≈ 1. Recalling the definitions of *x*_*h*_ and *y*_*h*_, the hair tip locations are given by

2.10a $${\tilde{x}}_{h,e}={\tilde{x}}_{h,0}+{\tilde{L}}_{h}\sin(\theta_{h,e})\approx {\tilde{x}}_{h,0}$$

and

2.10b $${\tilde{y}}_{h,e}={\tilde{y}}_{h,0}+{\tilde{L}}_{h}\cos(\theta_{h,e})\approx {\tilde{y}}_{h,0}+{\tilde{L}}_{h}.$$

Furthermore, suppose that the initial locations of the hair bases are such that

2.11a $${\tilde{x}}_{h,0}-{\tilde{x}}_{i,0}∼\delta (x_{h,0}^{*}-x_{i,0}^{*})$$

and

2.11b $${\tilde{y}}_{h,0}-{\tilde{y}}_{i,0}∼\delta (y_{h,0}^{*}-y_{i,0}^{*}),$$

where *h* ≠ *i,* i.e. the hairs are placed along an almost flat substrate with an unknown, scaled spacing of order *δ*. Substituting the above into (2.9), we obtain

2.12 $$\theta_{h,e}=\frac{{\tilde{L}}_{h}{\tilde{q}}_{h}}{{\tilde{S}}_{h}}\frac{K}{\delta^{2}}\sum_{i\neq h}{\tilde{q}}_{i}\frac{x_{h,0}^{*}-x_{i,0}^{*}}{{\left({\left(x_{h,0}^{*}-x_{i,0}^{*}\right)}^{2}+{\left(y_{h,0}^{*}-y_{i,0}^{*}+(1/\delta ){\tilde{L}}_{h}-(1/\delta ){\tilde{L}}_{i}\right)}^{2}\right)}^{3/2}},$$

with the parameters ${\tilde{L}}_{h}$, ${\tilde{q}}_{h}$, ${\tilde{S}}_{h}$, $x_{h,0}^{*}-x_{i,0}^{*}$, $y_{h,0}^{*}-y_{i,0}^{*}$ expected to be order unity for *h* = 1, 2, …, *N*.

The result in (2.12) indicates two conditions under which *θ*_*h*,*e*_ remains small. Firstly, *K*/*δ*^2^ is small and $1/\delta ({\tilde{L}}_{h}-{\tilde{L}}_{i})$ remains comparatively small. This is shown in figure 3, where the cases with relatively small values of *K* and/or large values of *δ* show little hair–hair interaction in terms of the equilibrium positions and contour shapes. Thus, either (i) *K* must be small compared with 1/*δ*^2^ or (ii) 1/*δ*^2^ is small overall.

Secondly, *θ*_*h*,*e*_ remains small when ${\tilde{L}}_{h}-{\tilde{L}}_{i}>1/\delta$ and dominates the denominator within the summation. In this case, (2.12) becomes

2.13 $$\theta_{h,e}\approx \frac{{\tilde{L}}_{h}{\tilde{q}}_{h}}{{\tilde{S}}_{h}}\sum_{i\neq h}{\tilde{q}}_{i}\frac{K\delta (x_{h,0}^{*}-x_{i,0}^{*})}{{\left({\tilde{L}}_{h}-{\tilde{L}}_{i}\right)}^{3}},$$

with $y_{h,0}^{*}=O(1),\;h=1,2,\ldots ,N$. Thus, for a given *Kδ* there is a value at which the comparative difference in hair length reduces the strength of the hair–hair interaction. In this instance, the equilibrium positions and angles tend to zero as *δ* becomes small.

#### 2.5.2. Small hair spacing

In §2.4, it was observed that the equilibrium angle tends to a limit set by *K* as *δ* became small (figure 4). Investigating further, suppose that *δ* diminishes to zero such that (2.11*a*) and (2.11*b*) each become zero and that *θ*_*h*,*e*_ is now some non-zero constant. Following the definitions of ${\tilde{x}}_{h,e}$, ${\tilde{y}}_{h,e}$, (2.9) becomes

2.14 $$\theta_{h,e}=\frac{{\tilde{L}}_{h}{\tilde{q}}_{h}}{{\tilde{S}}_{h}}K\sum_{i\neq h}{\tilde{q}}_{i}\frac{{\tilde{L}}_{i}\sin(\theta_{h,e}-\theta_{i,e})}{{\left({\tilde{L}}_{h}^{2}+{\tilde{L}}_{i}^{2}-2{\tilde{L}}_{h}{\tilde{L}}_{i}\cos(\theta_{h,e}-\theta_{i,e})\right)}^{3/2}},$$

which provides the limiting equilibrium positions of *N* hairs. It is expected that ${\tilde{L}}_{h},{\tilde{q}}_{h}$, ${\tilde{S}}_{h}$, ${\tilde{L}}_{h}$, $x_{h,0}^{*}$, $y_{h,0}^{*}$ are order unity for *h* = 1, 2, …, *N*, while *K* may vary in its order of magnitude and sets the limit of the equilibrium angles.

For an example, consider the two-hair case presented in figure 3, where the parameters are the same for both hairs (${\tilde{L}}_{h}=\tilde{L},{\tilde{S}}_{h}=\tilde{S},{\tilde{q}}_{h}=\tilde{q},h=1,2$). The hair responses are symmetric such that *θ*_1,*e*_ = −*θ*_2,*e*_, hence (2.14) becomes

2.15 $$\theta_{1,e}=\frac{{\tilde{q}}^{2}}{\tilde{S}\tilde{L}}K\frac{\cos(\theta_{1,e})}{4\sin^{2}(\theta_{1,e})}.$$

This provides the maximum equilibrium deflection of the two hairs. A similar process can be followed for more hairs and with non-equal parameters.

The analytical results above indicate that *K* and *δ* are the most important parameters in determining the extent of hair–hair interaction and the qualitative dynamics of the system.

### 2.6. Numerical examples of two different length hairs

**Figure 5.**
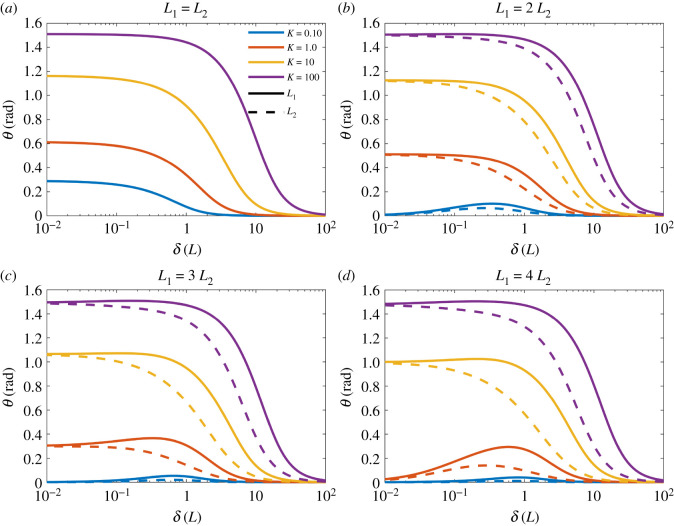
The effect of differing length on the equilibrium position of two hairs. The hair spacing is *δ* ∈ [10^−2^, 10^2^] and *K* = 0.1, 1, 10, 100 (blue, red, yellow, purple, respectively). (*a*) *L*_1_ = *L*_2_, (*b*) *L*_1_ = 2 *L*_2_, (*c*) *L*_1_ = 3 *L*_2_, (*d*) *L*_1_ = 4 *L*_2_. The total hair length in each case is *L*_1_ + *L*_2_ = 2.

Next, we investigate the influence of differing hair length. In figure 5, the equilibrium angles of two hairs are presented for four different ratios of hair lengths. The total hair length in the system is constant, with *L*_1_ + *L*_2_ = 2, to consider the same total resource.

The same trends and behaviours in the identical hair case (figures 4 and 5*a*) are seen in the non-identical cases with the equilibrium angles of the two hairs tending to the limit set by *K* as *δ* diminishes. For intermediary values of *δ*, the hairs have differing equilibrium angles from one another, which increase in disparity with the length ratio. In the bottom right panel (figure 5*d*) for *L*_1_ = 4*L*_2_ the small equilibrium angle case for ${\tilde{L}}_{h}-{\tilde{L}}_{i}>1/\delta$ (as detailed in §2.5.1) is seen when *K* = 0.1 and *K* = 1 as *δ* becomes small. The two hairs initially deviate from each other with notable equilibrium angles and both eventually diminish to zero as the values of ${\tilde{L}}_{i}-{\tilde{L}}_{j}$ become significant compared with *Kδ*.

**Figure 6.**
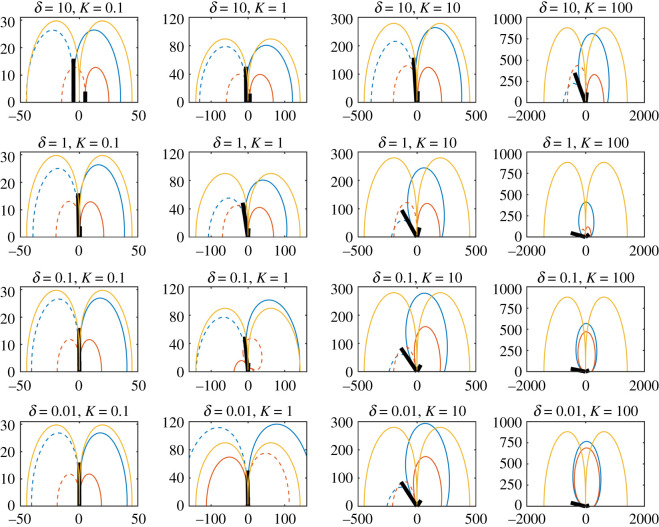
Sensitivity contours for two hairs of length *L*_1_ = 4 *L*_2_ (blue and red contours) from (2.9) and, for comparison, a single hair of length *L* = 2 (yellow contours). Contours show locations of an external point charge *q*_*p*_ = 10 that deflect hairs by at least 0.001 rad from their equilibrium positions for *δ* = 10, 1, 0.1, 0.01 (from top to bottom) and *K* = 0.1, 1, 10, 100 (from left to right). The horizontal unit is *x*_*p*_/*L* and the vertical unit is *y*_*p*_/*L,* with both measured in hair lengths. Blue curves relate to the left hair and red curves relate to the right hair. The black lines show the equilibrium position of the hairs (not to scale).

Examining further, the sensitivity contours of two hairs where *L*_1_ = 4*L*_2_, *L*_1_ + *L*_2_ = 2 are shown in figure 6 (blue contours relate to *L*_1_ located on the left-hand side, red contours relate to *L*_2_ on the right-hand side). Overlaid for comparison are the contours for a single hair of length 2 (yellow). The sensory contours again indicate the locations of an external point charge that deflect a hair by 0.001 rad.

The general trends in figure 3 continue to hold here with the two hairs acting as independent sensors when *δ* is large and *K* is small. As *K* increases (moving left to right) the interaction between the hairs grows, as shown by the change in the geometry and features of the contours. Additionally, for decreasing *δ* the hair–hair interaction also increases in line with previous results.

Comparing the two-hair system with the single hair, in most cases the single hair has a greater range of detection, especially when significant hair–hair interaction leads to large equilibrium angles in the two-hair system. In each case, the red contours are almost fully covered by the blue contours and hence appear to be of little benefit. Nonetheless, the smaller hair may, for example, act as a proximity sensor with the combination of hair deflections from the two hairs helping to indicate the distance of a point charge from the hairs. Furthermore, when significant hair–hair interaction occurs (*K* = 10, 100) the blind spot of the single hair is removed (the region above the single hair where the range of detection is significantly reduced). Additionally, the increased asymmetry in the system leads to a preferential direction of sensing.

There is one further phenomenon of interest. Consider the cases with *K* = 1, as *δ* becomes small substantial hair–hair interaction begins to occur. When *δ* decreases further the equilibrium position of the hairs begins to tend back to the resting position (as shown in figure 5). Yet, the results for *δ* = 0.1 and *δ* = 0.01 look remarkably different from cases with similar equilibrium angles (compare *δ* = 0.01, *K* = 0.1 with *δ* = 0.01, *K* = 1).

Indeed, for *δ* = 0.01, *K* = 1 the contours for the larger hair (blue) are now of a larger magnitude than the single-hair case. Thus, we see the amplification of a hair’s detection range owing to the adjacency of another shorter hair. This is an example of hair–hair coupling that leads to a positive synergy. Furthermore, the direction of the contours for the second hair (red) have reversed with the hair deflecting positively for point charges in the positive *x*-direction and negatively for point charges in the negative *x*-direction. Together these dynamics would have important biological and mechanical implications when considering detection capabilities and the sensitivity of hair arrays. Similar positively synergistic dynamics may be seen for a wider range of parameter values if the scales in (2.13) hold.

## 3. Sensory metrics and hair coupling

To understand these systems further, we now evaluate the sensory impact of hair–hair interaction from both the mechanical and biological perspectives through a range of metrics for the two identical hair system (§2.4). In particular, we consider electrostatic coupling in terms of the effect that a neighbouring hair has on the response of a hair of interest and quantify this in comparison with an isolated hair.

For mechanosensory hairs, the angle of hair deflection is widely considered to be the key sensory metric; however, depending on the mode and nature of the stimulus other metrics are also important. For example, in the case of aerodynamic sensing, typical metrics include the periodic response of mechanoreceptor hairs to oscillating far-field flows [11] and, within arrays, the aerodynamic hair–hair coupling [10,12]. Regarding electrostatic sensing, no previous work on mechanosensors has considered the key mechanics of this sensory modality and therefore there are no established metrics. Thus, informed by §2 and the aerodynamic literature there are four aspects of mechanosensor systems that we seek to understand in detail: sensitivity, directionality, location detection and electrostatic coupling.

### 3.1. Total area of coverage

**Figure 7.**
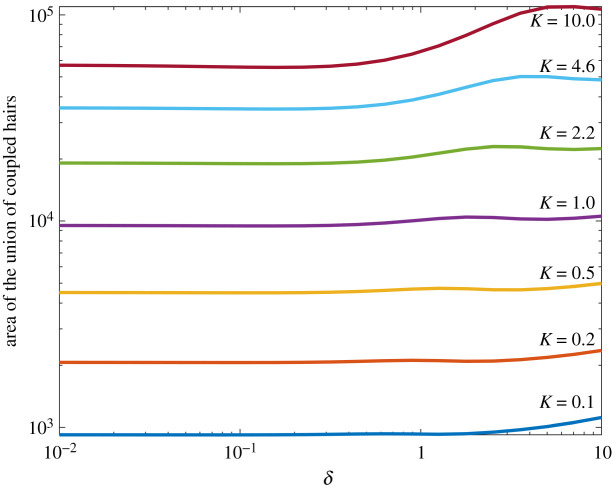
The total sensory area (where at least one hair deflects to at least the sensory threshold) of the coupled two-hair system. Hair spacing *δ* ∈ [10^−2^, 10^2^] and *K* ∈ [0.1, 10].

The first metric we consider is the total area covered by the sensitivity contours, as shown in figure 7. This measures the coverage of the multi-hair electromechanoreceptor system and represents the overall area in which at least one hair deflects to at least the sensory threshold for *q*_*p*_ = 10. Both hairs are of the same length and values of *K* = 0.1, 0.22, 0.46, 1.0, 2.2, 4.6, 10 (covering seven logarithmic intervals between 0.1 and 10) and *δ* ∈ [0.01, 10] are considered. The units of the axes here are *x*/*L* and *y*/*L* and thus the area is scaled by *L*^2^.

When *K* increases, the magnitude of the detection area increases owing to the greater sensitivity of the hairs. However, as the spacing between hairs decreases and coupling increases the detection area reduces in each case, and most strongly when *K* is large. Hence, when *δ* is sufficiently small there is an inhibitive effect on the sensory range of the system. Our results invoke two possible reasons for this (figure 3). Firstly, there is a change in the shape of the contours, and secondly, with the increased electromechanical sensitivity, both hairs are more strongly affected by both the presence of the point charge and the other hair response.

### 3.2. Overlapping sensory contours

**Figure 8.**
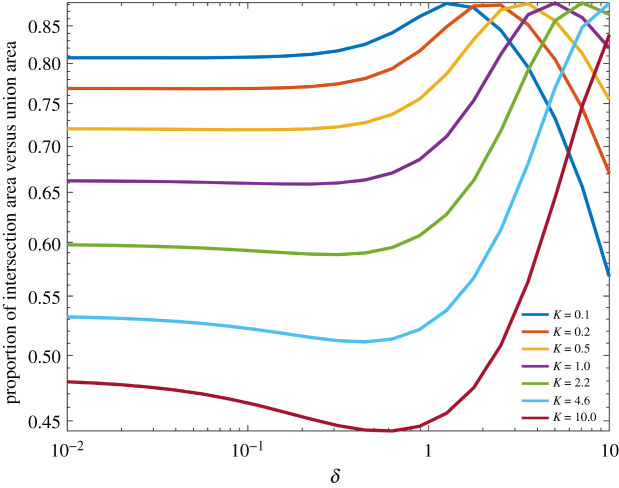
The proportion of the overlapping area (where both hairs deflect to at least the sensory threshold) to the total sensory area for the coupled two-hair system. Hair spacing *δ* ∈ [10^−2^, 10^2^] and *K* ∈ [0.1, 10].

The total sensory space divides into two regions: (i) where only a single hair can sense a given point charge and (ii) where both can. Thus, as the charge or the arthropod moves, the two-hair electromechanical sensory system can provide information about how the relative position of the point charge has changed within the arthropod’s sensory envelope depending on which hairs deflect and when. Therefore, the next metric we consider is the ratio of the area of overlap between the sensitivity contours of two hairs (the area in which both hairs deflect to at least the sensory threshold) to the total sensory area (figure 8). Both hairs are of the same length and *K* = 0.1, 0.22, 0.46, 1.0, 2.2, 4.6, 10 and *δ* ∈ [0.01, 10]. This metric provides a quantitative evaluation of how the sensory dynamics of a two-hair system changes with the hair spacing. Functionally, a system in which there is a large overlap between hairs provides a greater region within which it is feasible to determine the location and magnitude of a point charge.

When *K* increases the proportion of the total detection area covered by both hairs decreases overall. However, for each *K* there is a value of hair spacing at which a maximum in the ratio is achieved. The peaks for each *K* occur at the values of *δ* where the hairs are as close as possible without the equilibrium positions varying from the resting positions. For an example of this, see figure 3 and compare the cases where *K* = 0.1 as *δ* varies. The case when *δ* = 1 is near to the maximal proportion of overlap in the corresponding curve of figure 8. Values of *δ* that are larger than the peak values have a significantly smaller intersection area, while values of *δ* smaller than this value tend to a limit. For smaller *K*, the peak values associate with smaller values of *δ*.

From figure 3, we see that the results in figure 8 vary with the strength of coupling in the system and the equilibrium positions of the hairs. Hence, this metric shows that there is a threshold distance at which the hair–hair coupling becomes significant and begins to affect the sensory dynamics.

### 3.3. Directional ratio of sensory area of a coupled hair versus an uncoupled hair

There are several beneficial mechanical and geometric properties of the contours and hair–hair interaction that are not captured by the previous metrics. For example, figure 3 points to a breakdown of the symmetry of the contours about the hair and thus a change in directional sensing.

**Figure 9.**
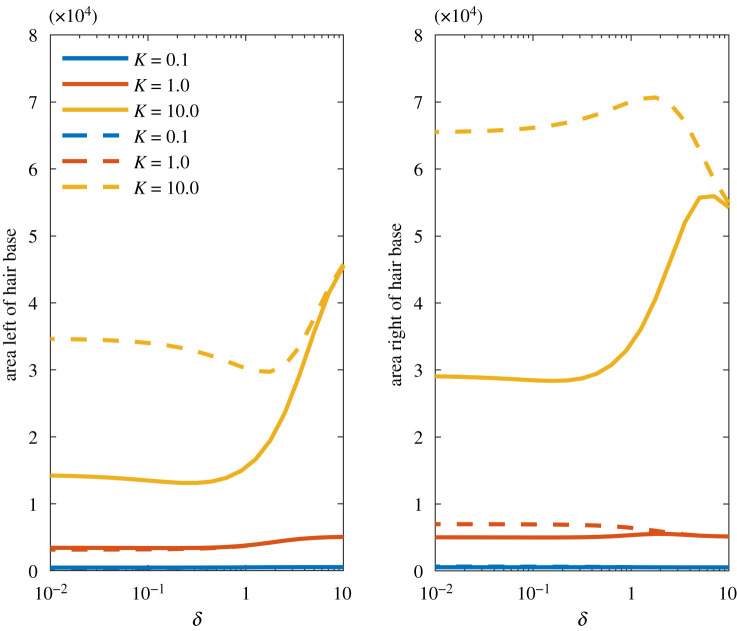
The sensory area to the left and right of the hair base for the left-hand hair within the coupled case (solid lines) and an uncoupled hair where the resting position is equal to the equilibrium position of the coupled hair (dashed lines). The hair spacing varies by *δ* ∈ [10^−2^, 10^2^] and *K* ∈ [0.1, 10].

Figure 9 presents the sensory area to the left and right of a hair. We compare the sensory area of the left-hand hair in the coupled two-hair system with the sensory area covered by a single uncoupled hair. The resting position of the uncoupled hair is set to equal the equilibrium position of the coupled hair to ensure that the results are not a result of geometry, but of hair–hair coupling. Notably, locations within the area to the left or right of the hair include regions that may induce positive and negative hair deflections. Only one of the two hairs needs consideration because of symmetry.

Considering the two-hair system as a whole, for large values of *δ* the coupling of the hairs is low and the sensory areas are close to the single-hair areas. As *δ* reduces and coupling increases, the sensory areas diminish nonlinearly with *K*. Thus, weak coupling may reduce the range of the system significantly and diminish a hair’s electromechanical sensitivity. Furthermore, with increased *K* there is a sensory preference to the right of the hair (positive *x*-direction) in both cases. For the uncoupled hair, this is solely due to the change in the orientation of the hair. For the coupled hair, the presence of a neighbouring hair has a distinct influence on the sensory capacity of the hair in both directions when *K* is very large, with a strong effect on deflections to the right of the hair.

These results point to two effects of coupling. Firstly, when the equilibrium position of the coupled hair varies greatly from the resting position the change in geometry leads to an asymmetry in the hair response and a directional preference. Secondly, the presence of the neighbouring hair, with sufficient coupling, may have an inhibitive effect on the sensory range of the hair, in all directions.

### 3.4. Coupling parameter under electrostatic stimulus

It remains unclear whether a neighbouring hair contributes enhancing or inhibiting coupling. Within the aerodynamic literature a coupling parameter, *κ*, is used to determine the nature and effect of coupling within the system [10,12], giving a measure of how much an isolated hair’s response changes when in the presence of another hair. The parameter *κ* is defined as

3.1 $$\kappa =\frac{\theta_{uc}-\theta_{c}}{\theta_{uc}},$$

where the subscripts *uc* and *c* refer to an uncoupled hair and coupled hair, respectively. A negative value of *κ* indicates an enhancing effect while a positive value indicates an inhibiting effect.

**Figure 10.**
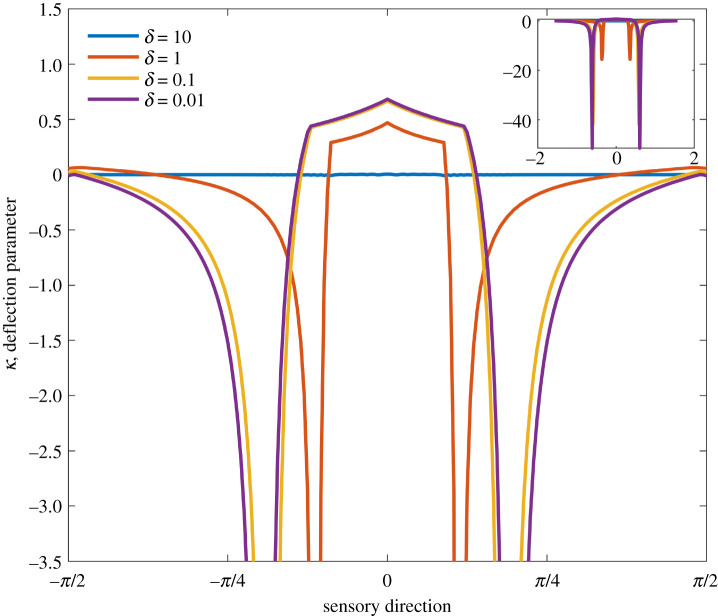
The coupling parameter for the union of hairs in the coupled system. The coupling parameter compares the expected deflection of a single uncoupled hair with the two-hair system for point charges at angles between −*π*/2 and *π*/2 from the centre of the hair array. The hair spacing varies as *δ* ∈ {10^−2^, 10^−1^, 0, 10} and *K* = 1.

This parameter can be employed to characterize the coupling in the electrostatic regime, presented in figure 10. The coupling parameter is calculated for the union of the two-hair coupled system in comparison with the union of a two-hair uncoupled system where the resting positions are rotated to the coupled equilibrium positions.

The contours in figure 3 show the locations $\left({\tilde{x}}_{p},{\tilde{y}}_{p}\right)$ at which deflections of 0.001 rad occur for the coupled hair system in §2.4. We can then use these values ${\tilde{x}}_{p},{\tilde{y}}_{p},q_{p}$ to find the corresponding values of *θ*_*uc*_ for the uncoupled hair system. This metric therefore measures the influence of coupling between hairs on the directional sensitivity of each hair in comparison with isolated hairs.

The point charge locations are quantified by a sensory direction defined as the angle between a point charge and the middle of the hair array taken from the vertical and range between −*π*/2 and *π*/2 about the origin. Values of *δ* = 10, 1, 0.1 and 0.01 are considered for *K* = 1 since the sensory areas are similar in figure 9 and there is little coupling for large *δ* yet significant coupling when *δ* is very small. For each direction of detection, the minimum value of *κ* for the two hairs is taken as the value of the coupling parameter.

From figure 10, scenarios where coupling is strong, e.g. *δ* < 10, negative values of *κ* are seen for the majority of detection angles. Other than for a small region in the parameter space above and nearly aligned with the deflected hairs, coupling has a beneficial effect on the sensitivity of the system. Indeed, when the coupling is strong with large *K*, similar results for the coupling parameter of the union of the system are seen (not shown). Of note, there are two large negative values for *κ* between −*π*/4 and 0 and 0 and *π*/4. These indicate the blind spots of the isolated hair system and hence show a further benefit of multiple hairs and the coupling between them.

## 4. Discussion and conclusion

We have investigated the mechanics of two-dimensional electromechanosensor hair arrays in the presence of a single point charge, including the Coulomb interaction between neighbouring hairs. We derived the equations generating sensitivity contours for arrays of *N* hairs for a chosen sensory threshold of hair deflection.

Within multi-hair arrays, our main topic of interest has been the electrostatic interaction and coupling of hairs considered in both the absence and presence of an external point charge. We have analysed how the strength of coupling within the system affects the qualitative and quantitative behaviour of the hairs through several numerical examples and metrics. The non-dimensionalization of the system ensures that the results and conclusions presented hold across scales and for different choices of the deflection threshold when the regime of the harmonic oscillator remains linear.

### 4.1. Electromechanical sensitivity

Regarding the influence of the parameters *K* and *δ*, our analysis shows that the parameters strongly affect the coupling in the system, producing increased coupling for larger values of *K* and small values of *δ*. Notably, *K* determines the electromechanical sensitivity of the hair system and therefore the range of detection for each hair and the overall magnitude of hair–hair coupling. Meanwhile, *δ* modulates the level of electrostatic coupling such that, for sufficiently large *δ,* electrostatic coupling may be negligible despite the value of *K*.

As discussed in §2.6, in some instances (e.g. when significant physical asymmetry occurs between the hairs), hair–hair coupling can dramatically amplify the detection range of a hair array. The sensory implications of this novel phenomenon of electrostatic coupling may be wide-reaching and merits empirical investigation.

### 4.2. Electrostatic coupling

Electrostatic coupling has been shown to affect the mechanical behaviour of hairs in two ways. Firstly, it determines the equilibrium position of the hairs. These positions qualitatively and quantitatively influence the sensitivity contours by changing the overall shape of the coupled system. Secondly, with increased coupling, the response of each hair to others becomes increasingly influential.

To quantify and evaluate electrostatic coupling within arrays of hairs, we applied several metrics that assess the overall sensory effects of coupling. In general, these results point to significant inhibitory coupling regarding the range of the sensory system for large values of *K* (relative to uncoupled or less coupled systems), yet strong enhancing coupling regarding directionality and overall sensory coverage (as measured by the coupling parameter *κ* in figure 10). These aspects of coupling are the result of the change in geometry of the system and the interaction between hairs.

By way of comparison, the single isolated hair was shown to often out-compete the two-hair system with respect to the range and area of detection, especially when significant coupling occurred. However, there are several biological and mechanical advantages to multiple hairs. For example, in the single-hair system, there is a large area above the hair in which its ability to detect a point charge significantly diminishes. However, for a multi-hair system, this sensitivity loss can be overcome, providing an arthropod with a more uniform coverage above the body surface.

Furthermore, it has previously been shown that arrays of mechanosensory and filiform hairs can provide an animal with several sensory capabilities that an individual hair cannot display (e.g. in spiders [42], and in crickets [9]). Indeed, arrays may have significant variation in the lengths, geometries and characteristics of the individual hairs within. The diversity of hairs may provide several sensory benefits, e.g. frequency preference or electrical charge, simply through a change in hair length.

As shown in [22], when both the aerodynamic and electrostatic hair responses are considered, variation in hair length may lead to increased sensitivity and specificity within either modality. When arrays of hairs are considered, the benefits of different hair lengths become more apparent in both the collective capacity to detect certain stimuli (e.g. differences in total charge or frequency tuning) and the synergistic relationships between hairs through electrostatic coupling (as shown above) and viscous-mediated coupling [10,12]. Hence, a diversity of hair lengths enhances the sensory capacity of an arthropod by increasing the breadth of stimuli that it may sense and by providing the ability to differentiate between stimuli. Finally, future investigations are required to quantify the comparative effects of viscous-mediated coupling and electrostatic coupling within an array and classify when their effects are enhancing or inhibitory.

### 4.3. Detection of location and magnitude of charges

The sensitivity contours in figure 1*d* also illustrate how electromechanosensory arrays can locate charges in the environment. Consider the situation where both hairs experience a deflection of $\theta_{s}=-0.001\;\mathrm{rad}$ due to a point charge of unknown magnitude *q*_*p*_ and unknown location. From figure 1*d*: if *q*_*p*_ = 1 the charge is located at the intersection of the solid blue contours; if *q*_*p*_ = 2 it is at the intersection of the solid red contours, etc. Thus, even if the magnitude of the charge is unknown, some information about its location can be deduced, namely that it lies on the curve joining the intersections of the pairs of homochromatic solid contours in figure 1*d*. With more than two hairs, more precise information can be obtained, about both the location of the charge and its magnitude; for example, a three-hair system can pinpoint both the location and magnitude of a point charge (for a three-dimensional domain a four-hair system can do the same). The detection capabilities of the system vastly increase with additional hairs. Hence, it is possible for an arthropod to map its electrostatic environment in real time from multidirectional hair arrays distributed around its body. This proposition warrants empirical investigations of arthropods endowed with such a hair array and demonstrable aerial electroreception.

### 4.4. Outlook

Currently, evidence for the presence and strength of hair–hair electrostatic interactions within an array in biological systems remains elusive. However, the analysis presented above is instructive and highlights ways forward.

Firstly, the analysis highlights the potential role of electrostatic coupling in hair systems and its possible impact on their dynamics and sensory performance (even for mildly charged hairs). The results presented in this paper show that, in nature, electrostatic coupling and hair–hair interaction are feasible over a large range of biologically relevant *K* values. Therefore, electrostatic coupling is expected to play a significant role in the mechanical response of filiform hairs at the scale of the array and over the whole body.

Secondly, the results highlight the impact of coupling and its influence within a system despite the changes in geometry (e.g. the case where the two hairs are of different lengths). When the difference in hair lengths was sufficient, the equilibrium positions of the hairs tended to zero, yet the effect of coupling was still strong. If higher values of *K* can be maintained in practice (through the clustering of several hairs, or curved geometries) and hair equilibrium positions are close to their resting positions, similar interesting synergistic coupling may occur. Furthermore, the capacity for electrostatic interactions and sensing considered here unveils new and important consequences for aerodynamic and acoustic stimulus detection and opens up the possible multi-modality of these mechanosensory systems. Indeed, the consideration of electrical sensing and electrostatic coupling may well further our understanding of the morphological diversity of filiform hairs, the density of their arrays and, not least, other sensory processes that are carried out by these hairs (e.g. hearing, fluid flow sensing and proprioception).

Thirdly, our findings hold for other charge distributions such as a dipole or a uniform charge distribution. The hair response due to the point charge for these alternative distributions will be the same as the unipolar case studied here, up to a constant (e.g. a different strength of the electrical field would be required to produce the same magnitude response). However, the charge distribution will significantly impact coupling in the system, changing its level and nature at given values of *K* and *δ*. Nonetheless, the overall qualitative impact that coupling has will be the same as studied here (i.e. similar positive and negative synergistic effects that enhance or inhibit the sensory capacity of the system).

Finally, to clarify the resultant effects of coupling, the majority of the numerical analyses have focused on two-hair systems. The understanding of coupling in large arrays has not been covered. The analysis of large arrays interacting with moving charges will further inform the design constraints observed in real arthropod filiform hair fields and their sensory envelopes. Yet, such work is not without difficulty given the possible large morphological diversity that arthropods have evolved to acquire information from their environment.

## Data Availability

All scripts used in this study are openly accessible through https://github.com/StochasticBiology/boolean-efflux.git. The data are provided in the electronic supplementary material [43].
